# Botanicals as a zinc oxide alternative to protect intestinal cells from an *Escherichia coli* F4 infection *in vitro* by modulation of enterocyte inflammatory response and bacterial virulence

**DOI:** 10.3389/fvets.2023.1141561

**Published:** 2023-03-09

**Authors:** Andrea Bonetti, Andrea Piva, Ester Grilli

**Affiliations:** ^1^Dipartimento di Scienze Mediche Veterinarie, Università di Bologna, Bologna, Italy; ^2^Vetagro S.p.A., Reggio Emilia, Italy; ^3^Vetagro Inc., Chicago, IL, United States

**Keywords:** piglets, weaning, post-weaning diarrhea, *Escherichia coli* F4, zinc oxide, essential oils, natural extracts, botanicals

## Abstract

Pharmacological doses of zinc oxide (ZnO) have been widely used in pig industry to control post-weaning diarrhea (PWD) symptoms exacerbated by enterotoxigenic *Escherichia coli* F4 infections. Because of environmental issues and regulatory restrictions, ZnO is no longer sustainable, and novel nutritional alternatives to manage PWD are urgently required. Botanicals represent a wide class of compounds employed in animal nutrition because of their diverse beneficial functions. The aim of this study was to investigate the *in vitro* protective action of a panel of essential oils and natural extracts on intestinal Caco-2 cells against an *E. coli* F4 infection. Moreover, we explored the potential mechanisms of action of all the botanicals compared to ZnO. Amongst the others, thyme essential oil, grape seed extract, and *Capsicum* oleoresin were the most effective in maintaining epithelial integrity and reducing bacterial translocation. Their mechanism of action was related to the modulation of cellular inflammatory response, the protection of tight junctions' expression and function, and the control of bacterial virulence, thus resembling the positive functions of ZnO. Moreover, despite their mild effects on the host side, ginger and tea tree essential oils provided promising results in the control of pathogen adhesion when employed during the challenge. These outcomes support the advantages of employing selected botanicals to manage *E. coli* F4 infections *in vitro*, therefore offering novel environmentally-friendly alternatives to pharmacological doses of ZnO capable to modulate host-pathogen interaction at different levels during PWD in pigs.

## Introduction

Zinc oxide (ZnO) is a molecule widely employed at pharmacological doses in animal husbandry (1,000–3,000 ppm in complete feed) to treat post-weaning diarrhea (PWD). This condition mainly affects piglets, causing significant economic losses because of reduced animal growth performance, costs for treatments, and mortality (1). The onset of PWD is primarily linked to Enterotoxigenic *Escherichia coli* (ETEC) strain F4, which exploits piglets' weaning stress to target the gastrointestinal tract (2). ETEC overgrowth in the small intestine triggers the expression of several virulence genes, like bacterial adhesins or heat-labile and heat-stable toxins. Thus, pathogen easily adhere to enterocytes, where they secrete toxins to finally elicit the production of diarrhea (3). Studies demonstrated that *E. coli* F4 infection further impairs weaning stress by stimulating inflammation and reducing gut integrity in one of the most delicate phases of the pig production cycle (4–7).

It has been suggested that the reason why ZnO is particularly effective in managing PWD symptoms is related to its antimicrobial effect (8–10). However, whether it is true that ZnO possesses the capacity to reduce bacterial growth *in vitro*, several *in vivo* studies showed that antimicrobial effects against *E. coli* F4 are mild and limited to medicinal doses (11–13). Rather, the mechanism of action of ZnO seems broadly due to its antioxidant, anti-inflammatory, and anti-adhesive properties, together with its extensive supportive action on the intestinal mucosa (1, 14).

However, the large and prolonged utilization of pharmacological doses of ZnO in pig nutrition led to the onset of environmental issues and the acceleration of antimicrobial and heavy metal resistance spread amongst bacteria (15–17). For these reasons, European authorities decided to impose a ban on medicinal doses of ZnO (18), so novel alternatives to manage PWD and weaning stress are urgently required (19). The new substitutes should not only represent safe and sustainable ZnO replacements, but also embody the same multi-factorial properties that pharmacological ZnO exerts at the gut level.

A wide reservoir of potential novel molecules comes from nature, where a large number of bioactive compounds are innately synthesized by plants to protect themselves against pathogens and face stressful situations (20–22). Essential oils (EO) and powder extracts are widely studied in animal nutrition to support growth performance and control microbial growth (23, 24). Other than antibacterial activity, such compounds also exert anti-inflammatory and antioxidant actions, together with their capacity to enhance epithelial integrity and function at the intestinal level (25). All the effects of natural products are largely ascribed to the active principles they contain, such as polyphenols and terpenes (25, 26). The bioactive functions of these molecules generally follow the ones exerted by ZnO, so EO and extracts represent promising candidates to help piglets face weaning inflammation and stress, especially when triggered by *E. coli* F4 infections (1).

The aim of this study was to explore the protective effects of several EO and powder extracts during an *in vitro* infection by *E. coli* F4 on Caco-2 cells, a recognized model for intestinal studies (27). In particular, we tried to elucidate the potential mechanism of action of all the tested compounds, to identify the most effective to maintain enterocyte's monolayer integrity by modulating the inflammatory response, reducing cellular susceptibility to the pathogen, and affecting ETEC adhesive properties.

## Materials and methods

### Chemicals and reagents

Cell culture reagents and chemicals were obtained from Sigma-Aldrich (Milan, Italy), unless otherwise specified. Thyme essential oil (ThyEO, 48% thymol), *Capsicum* oleoresin (CapOR, 6% total capsaicinoids), ginger essential oil (GEO, 29–40% zingiberene, 10–14% sesquiphellandrene), and tea tree oil (TTO, >30% terpinene-4-ol, 5–13% α-terpinene, 10–28% γ-terpinene) were provided by Frey+Lau (Henstedt-Ulzburg, Germany). Grape seed extract (GSE, >80% total polyphenols as catechin equivalents at 280 nm—UV/VIS) and olive leaf extract (OE, 10% oleuropein) were obtained from Layn Natural Ingredients (Shangai, China). Zinc oxide (ZnO) was obtained from Alfa Aesar (Karlsruhe, Germany).

Stock solutions of all the bioactive compounds were prepared in ethanol 100% (v/v) and supplemented in culture medium at a final working concentration of ethanol ≤ 1% (v/v). Zinc oxide stock solution was prepared in 5% acetic acid (v/v) and supplemented in culture medium at a final working concentration of acetic acid ≤ 0.1% (v/v).

Final tested concentrations were 20 ppm for ThyEO, 100 ppm for GSE, CapOR, GEO, TTO, OE, and 0.2 mM for ZnO. For essential oils and extracts, the 100 ppm concentration was chosen basing on their effect on viability and their antioxidant potential previously assessed on unchallenged Caco-2 cells by our research group (28). ThyEO and ZnO concentrations differ from the others because higher doses were not tolerated by Caco-2 cells on 3.0 μm diameter Transwell^®^ inserts, as shown by our preliminary experiments (see Supplementary Figures 1, 2), in which high ThyEO and ZnO alone (without bacterial challenge) produced a considerable drop in TER.

### Cell line and culture conditions

The human colon carcinoma cell line (Caco-2) was obtained from DSMZ (Braunschweig, Germany). Cells were used at a passage between 10 and 20, and were routinary maintained at 37°C, 5% CO_2_ atmosphere, and 95% relative humidity. Basal maintenance medium was composed of Dulbecco's modified Eagle's medium (DMEM) high-glucose supplemented with 10% fetal bovine serum, 1% L-glutamine, 1% penicillin/streptomycin, and 1% non-essential amino acids.

### Bacteria and culture conditions

The bacterium employed in the study was a field strain of *Escherichia coli* K88/F4, expressing heat-labile and heat-stable toxins (LT^+^, STa^+^, STb^+^), and originally isolated from the intestine of a piglet with post-weaning diarrhea. Bacteria were routinely cultured in brain-heart infusion broth (BHI, WVR International, Milan, Italy) at +37°C for 24 h. Daily 1:100 passage was performed to maintain active cultures (29).

On challenge day, bacterial inoculum was prepared by making a 1:80 passage of the overnight culture in a fresh BHI tube. After 4 h, when bacteria reached the late-midlog phase of growth, bacterial turbidity was measured by assessing absorbance at 630 nm. The obtained turbidity was interpolated in an absorbance—CFU/mL growth curve previously prepared to obtain a precise bacterial count for the inoculum of the following experiments, as previously described by Roselli et al. (14).

### Infection of Caco-2 cells on porous filters

To measure Transepithelial Electrical Resistance (TER) and Bacterial Translocation (BT) during a bacterial challenge, Caco-2 cells were grown and differentiated for 30 days in 12 well plates on porous Transwell^®^ inserts (3.0 μm diameter pores) (Corning, Massachusetts, USA) (30).

Infection method was adapted from previously published studies (14, 31, 32). Briefly, on the challenge day, TER was measured using an epithelial tissue voltohmmeter (Millicell ERS-2, Merck, Darmstadt, Germany) to obtain basal TER values for each filter. Then, cells were washed twice with DPBS and apically infected with 5 × 10^7^ CFU/mL bacteria (multiplicity of infection of 100) in basal medium without P/S and supplemented with the various tested substances. Treatments were also included in the basolateral media, which consisted of basal media without P/S. Two controls were prepared: a negative control (CTR), without bacteria, and a positive control (CTR+), with only the bacteria.

At 2 and 4 h after the beginning of the infection, cellular integrity was monitored by measuring TER. At both timepoints, 100 μL of basolateral medium were collected from each filter and serially diluted in sterile saline. Then, aliquots of the most appropriate dilutions were seeded on BHI plates. After 24 h of incubation at +37°C, viable translocated bacteria were counted.

At the end of all the TER measurements, infection medium was removed, cells were washed twice with DPBS, harvested, and stored at −80°C until further processing for gene expression analysis.

### Gene expression of infected Caco-2 cells

Gene expression was performed according to previously published studies on similar *in vitro* models of Caco-2 cells (28, 30). Briefly, after thawing of the harvested samples, Caco-2 RNA was extracted using NucleoSpin RNA Kit (Macherey-Nagel, Düren, Germany) with DNase digestion according to manufacturer's instructions. RNA yield and purity was assessed by A230, A260, and A280 nm measurements at the spectrophotometer (μDrop Plate and Varioskan LUX, Thermo Fisher Scientific, Waltham, MA, USA).

RNA was then retrotranscribed with iScript cDNA Synthesis Kit (Bio-Rad Laboratories, Hercules, CA, USA) according to manufacturer's instruction. cDNA was subsequently diluted to 5 ng/μL and analyzed *via* qPCR analysis. Reactions were prepared using iTaq Universal SYBR Green Supermix (Bio-Rad Laboratories, Hercules, CA, USA) and analyzed through CFX96 Real-Time PCR Detection System (Bio-Rad Laboratories, Hercules, CA, USA) under the following conditions: 3 min at 95°C, followed by 40 cycles of 95°C for 10 s and 60°C for 30 s. The specificity of each reaction was evaluated by melting-curve analysis.

Gene expression was normalized using two reference genes, i.e., ribosomal protein lateral stalk subunit P0 (RPLP0) and glyceraldehyde-3-phosphate dehydrogenase (GAPDH). The relative changes in gene expression were calculated using the 2^−ΔΔCt^ method (33).

Table 1 displays forward and reverse primers of selected target genes, which were obtained from Merck (Darmstadt, Germany).

**Table 1:** Primers used in this study for gene expression analysis.

**Function**	**Gene**	**Sequences (5^′^ → 3^′^)**	**Product length (bp)**	**References**
Tight-junction integrity	*ZO-1*	F: CGGGACTGTTGGTATTGGCTAGA R: GGCCAGGGCCATAGTAAAGTTTG	184	(101)
	*ZO-2*	F: CTAGCAGCGATCAACTTAGGGACAA R: CCCAGGAGTTTCATTACCAGCAA	158	(101)
	*CLD-1*	F: GCACATACCTTCATGTGGCTCAG R: TGGAACAGAGCACAAACATGTCA	92	(101)
Innate immune response	*TNFα*	F: TCTCGAACCCCGAGTGACAA R: TATCTCTCAGCTCCACGCCA	124	(102)
	*IL-1β*	F: AATCTGTACCTGTCCTGCGTGTT R: TGGGTAATTTTTGGGATCTACACTCT	78	(103)
	*IL-8*	F: ATGACTTCCAAGCTGGC R: ACTTCTCCACAACCCT	174	(104)
	*BD1*	F: CCTACCTTCTGCTGTTTACTC R: ACTTGGCCTTCCCTCTGTAAC	186	(105)
Housekeeping genes	*RPLP0*	F: GCAATGTTGCCAGTGTCTG R: GCCTTGACCTTTTCAGCAA	142	(106)
	*GAPDH*	F: TGCACCACCAACTGCTTAGC R: GGCATGGACTGTGGTCATGAG	87	(107)

F, forward; R, reverse; ZO-1, zonula occludens 1; ZO-2, zonula occludens 2; CLD-1, claudin 1; TNFα, tumor necrosis factor α; IL-1β, interleukin 1β; IL-8, interleukin 8; BD1, beta defensin 1; RPLP0, ribosomal protein lateral stalk subunit P0; GAPDH, glyceraldehyde 3 phosphate dehydrogenase.

### Adhesion assay

As a marker of bacterial virulence, an adhesion assay was performed to assess bacterial ability to interact with target cells (14). Caco-2 cells were differentiated in 24 well plates; on the infection day, after two washes with DPBS, Caco-2 were infected with 5 × 10^7^ CFU/mL bacteria in basal medium without P/S and supplemented with the various tested substances. Two controls were prepared: a negative control (CTR), without bacteria, and a positive control (CTR+), with only the bacteria.

After 1 h of incubation, bacteria in suspension were eliminated by washing cells four times with DPBS. Then, Caco-2 were lysed with 0.5% Triton X-100 in DPBS for 10 min and, after serially diluting lysed cells in sterile saline, appropriate dilutions were seeded on BHI agar. After 24 h of incubation at +37°C, viable adhered bacteria were counted.

### Immunofluorescence assay

To assess the localization of zonula occludens 1 (ZO-1), a tight junction protein, during the bacterial challenge, Caco-2 cells were stained *via* an immunofluorescence assay. Cells were cultivated on glass coverslips maintained inside 6 well plates, with each well corresponding to a single group of treatment.

After differentiation, Caco-2 cells were washed twice with DPBS, and then infected with 5 × 10^7^ CFU/mL bacteria in basal medium without P/S and supplemented with the various tested substances. Two controls were prepared: a negative control (CTR), without bacteria, and a positive control (CTR+).

After 2 h of incubation, bacteria in suspension were eliminated and cells washed twice with DPBS. Then, Caco-2 were fixed with 4% paraformaldehyde in DPBS for 20 min. Subsequently, cells were permeabilized with 0.5% Triton X-100 for 15 min, and then blocked in 10% goat serum for 1 h. ZO-1 primary monoclonal antibody (ThermoFisher Scientific, Walthan, MA, USA) was diluted in DPBS containing 2% bovine serum albumin (BSA) and 0.05% saponins, and incubated on Caco-2 cells for 3 h at +4°C in a humidified chamber. After three washes with 0.2% BSA + 0.05% saponins in DPBS, secondary antibody conjugated to fluorescein isothiocyanate (FITC) (ThermoFisher Scientific, Walthan, MA, USA) was used to probe for 1 h the bounded primary antibody. Two washes were finally performed with DPBS supplemented with 0.2% BSA and 0.05% saponins to remove unbounded antibodies. Slides were mounted with Fluoroshield containing 4′,6-diamidino-2-phenylindole (DAPI), images acquired using a fluorescence microscope (Nikon Corporation, Tokyo, Japan), and pictures processed using NIS-Elements software (Nikon Corporation, Tokyo, Japan).

### Statistical analysis

Results are obtained from at least two independent experiments. For each experiment, the experimental unit was the well, with *n* = 6. Data are displayed on graphs as means ± SEM. For TER, BT, gene expression, and adhesion assays, data were analyzed using GraphPad Prism v.9.4.0 (GraphPad Software, Inc., San Diego, CA, USA) performing One-Way ANOVA analysis with Dunnett multiple comparisons test, comparing all the experimental groups with the mean of CTR+ group. Differences were considered significant when *p* ≤ 0.05, trends were identified when *p* ≤ 0.1.

## Results

### Epithelial integrity

**Figure 1:**
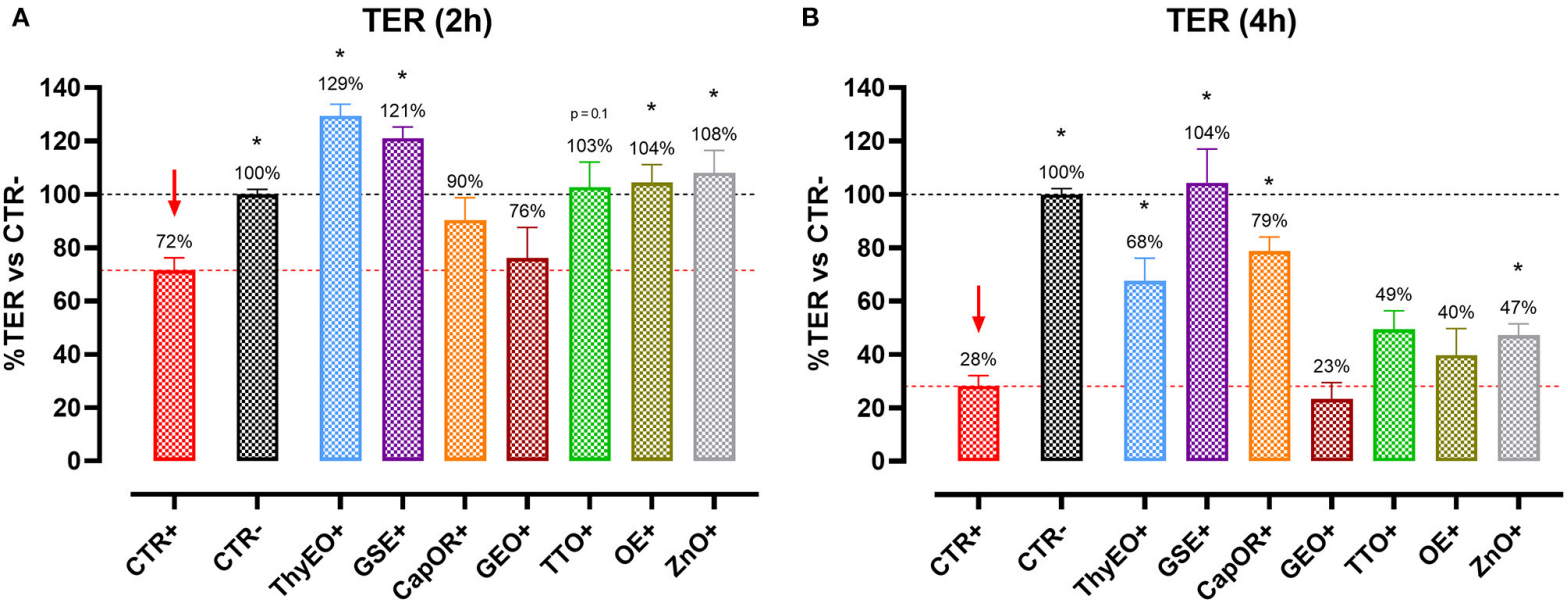
TER of Caco-2 cells treated with essential oils or powder extracts and challenged with *E. coli* F4 at 2 h **(A)** and 4 h **(B)** after the beginning of the infection. Groups with bacterial infection are represented with a “+” in the name. Data in the graphs are represented as means ± SEM; percentage values are referred to negative control (CTR–) for both the investigated timepoints. One-Way ANOVA analysis is performed against positive control (CTR+), identified with a red arrow; asterisks “*” denote significant differences with *p* < 0.05, while tendencies are highlighted by their *p*-value.

Figure 1 reports the TER measurements of Caco-2 cells treated with selected compounds during an *E. coli* F4 challenge. Results are presented as percentage of TER referred to the negative control group, considered as the normal TER value in a steady condition of culture.

Data show that the ETEC challenge could significantly reduce the integrity of the cellular monolayer at both 2 h and 4 h after the beginning of the infection. At 4 h, considerable damages on infected Caco-2 led TER to a value equal to 28% of the negative control.

Despite the presence of the challenge, several treatments were effective in protecting Caco-2 cells from the drop in TER exerted by *E. coli* F4. In particular, ThyEO and GSE kept TER at a level significantly higher than the positive control, with values even greater than the negative control at the first timepoint (129% for ThyEO+ and 121% for GSE+ groups). Moreover, CapOR limited the drop in TER that Caco-2 cells would have experienced at 4 h (79% of CapOR+ instead of 28% of CTR+). Also, ZnO significantly protected cells from the effects of the challenge, even if its action was considerable at 2 h (108%), but mild at 4 h (47%). Similar behaviors were registered also for TTO and OE, while GEO did not show significant improvements in TER at both timepoints.

### Bacterial translocation

**Figure 2:**
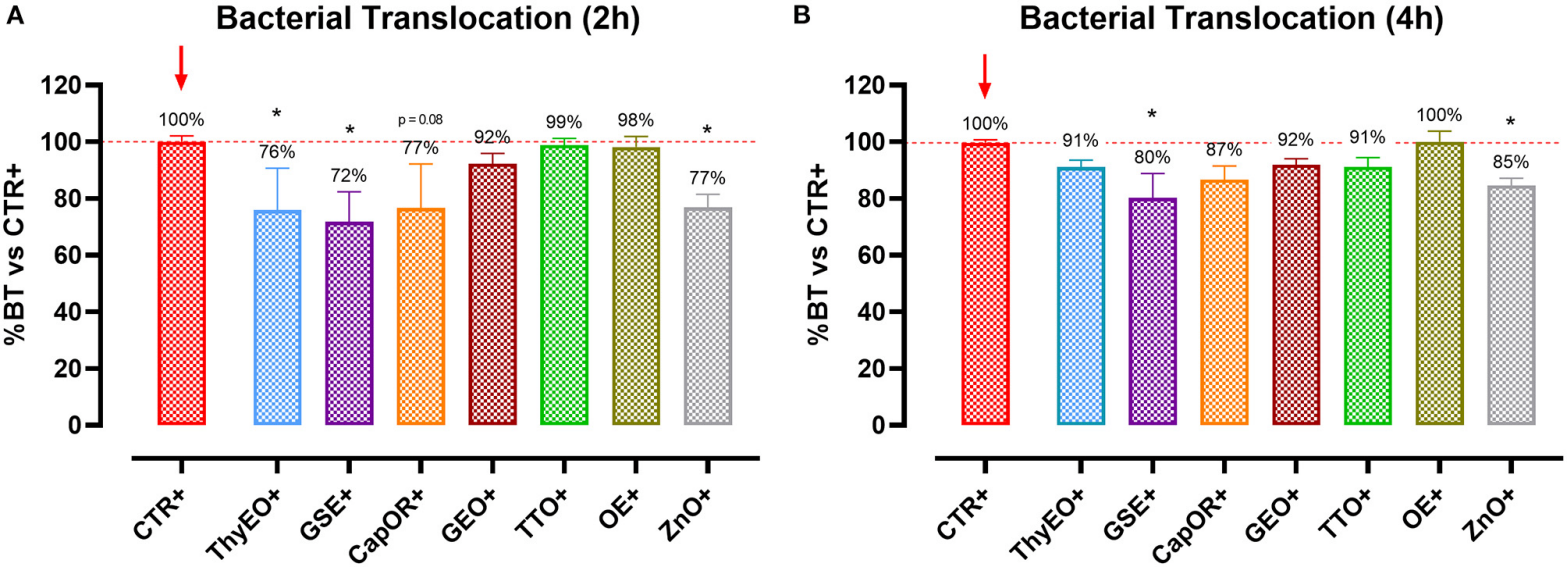
Bacterial translocation of *E. coli* F4 across Caco-2 cells treated with essential oils or powder extracts at 2 h **(A)** and 4 h **(B)** after the beginning of the bacterial infection. Groups with bacterial infection are represented with a “+” in the name. Data in the graphs are represented as means ± SEM; percentage values are referred to positive control (CTR+) for both the investigated timepoints. One-Way ANOVA analysis is performed against positive control (CTR+), identified with a red arrow; asterisks “*” denote significant differences with *p* < 0.05, while tendencies are highlighted by their *p*-value.

To understand the capacity of the tested bioactive compounds to limit *E. coli* F4 passage across the cellular monolayer, a bacterial translocation assay was performed. Results are presented in Figure 2 and expressed as a percentage of bacterial translocation referred to the value obtained at each timepoint by CTR+, considered the highest possible passage in our system.

Results show that ThyEO and GSE significantly reduced bacterial passage across Caco-2 cells at 2 h (−24 and −28%, respectively), with mean values numerically close to the ones of ZnO (77% of CTR+). A reduction trend was measured for CapOR, while other treatments, despite improving TER, did not significantly reduce bacterial translocation.

At 4 h differences are less evident, even if GSE significantly reduced *E. coli* F4 passage at the same extent of ZnO (−20 and −15%, respectively). Other treatments, like ThyEO, CapOR, TTO, and GEO, could only numerically reduce bacterial translocation, while OE did not help cells to avoid pathogen passage across the enterocyte monolayer.

### Gene expression of tight junctions and inflammatory markers

**Figure 3:**
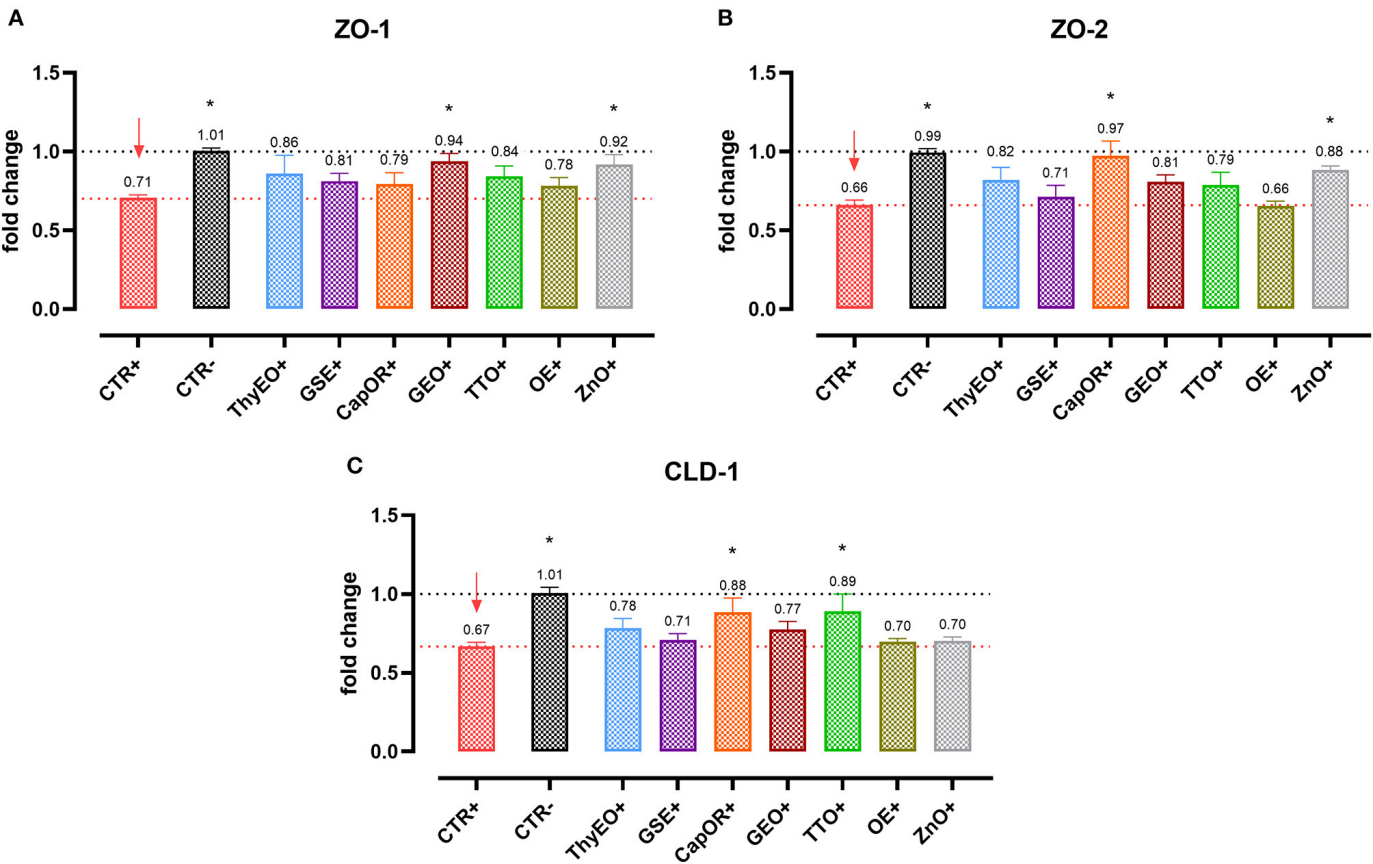
Gene expression analysis of Caco-2 cells treated with essential oils or powder extracts and challenged with *E. coli* F4 at 4 h after the beginning of the infection. The analyzed markers of cellular monolayer integrity are ZO-1 **(A)**, ZO-2 **(B)**, and CLD-1 **(C)**. Groups with bacterial infection are represented with a “+” in the name. Data in the graphs are represented as means ± SEM. One-Way ANOVA analysis is performed against positive control (CTR+), identified with a red arrow; asterisks “*” denote significant differences with *p* < 0.05, while tendencies are highlighted by their *p*-value.

**Figure 4:**
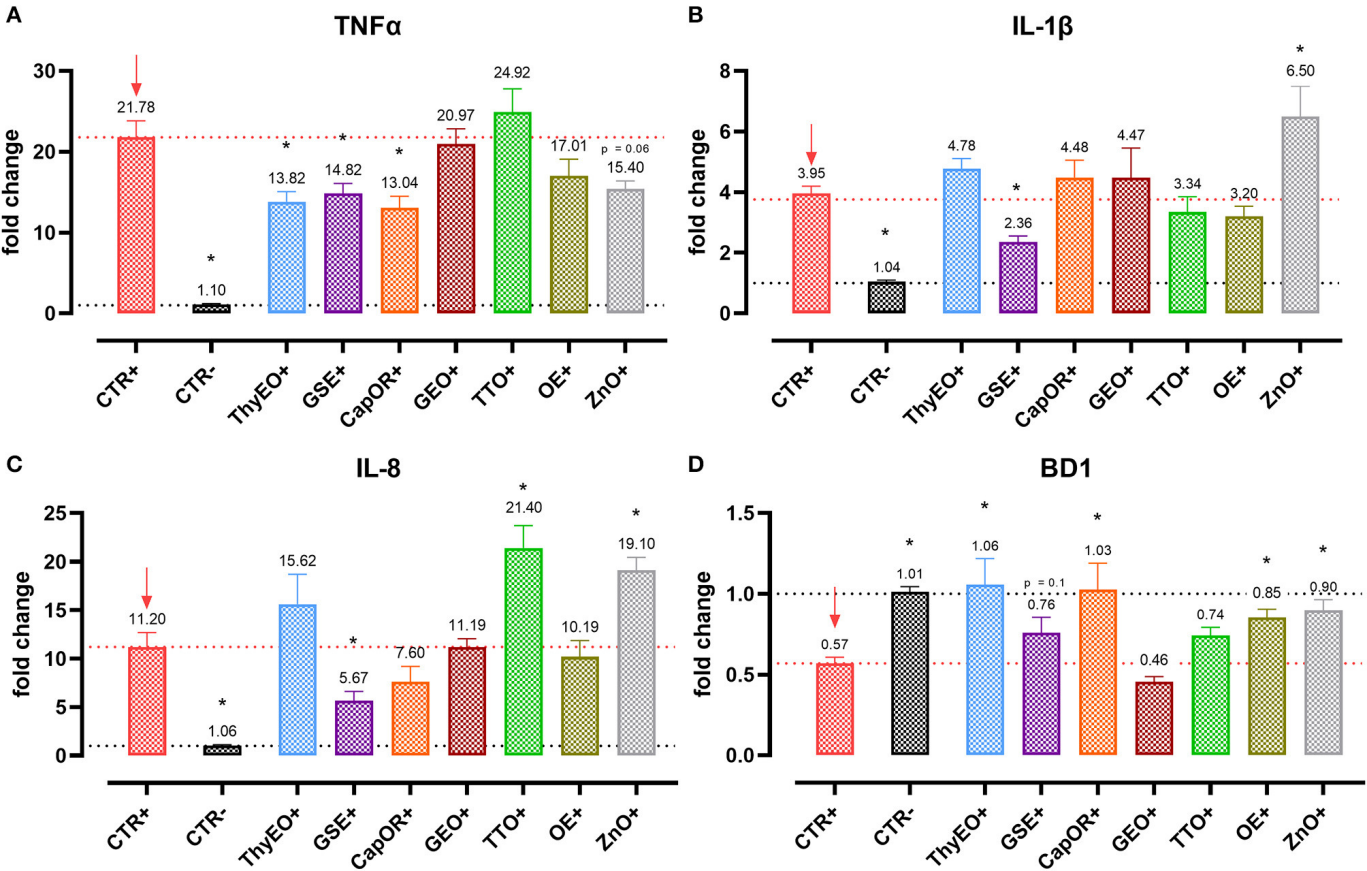
Gene expression analysis of Caco-2 cells treated with essential oils or powder extracts and challenged with *E. coli* F4 at 4 h after the beginning of the infection. The analyzed markers of innate immune response are TNFα **(A)**, IL-1β **(B)**, IL-8 **(C)**, and BD1 **(D)**. Groups with bacterial infection are represented with a “+” in the name. Data in the graphs are represented as means ± SEM. One-Way ANOVA analysis is performed against positive control (CTR+), identified with a red arrow; asterisks “*” denote significant differences with *p* < 0.05, while tendencies are highlighted by their *p*-value.

Infected cells were harvested and mRNA levels investigated to monitor the ability of the tested bioactive principles to modulate the expression of tight junctions (Figure 3) and innate immune response markers (Figure 4). Tight junction integrity was assessed by investigating the expression of zonula occludens 1 (ZO-1), zonula occludens 2 (ZO-2), and claudin 1 (CLD-1) genes, while immune response was evaluated by analyzing the expression of cytokines like TNFα, IL-1β, IL-8, and beta defensin 1 (BD-1).

*Escherichia coli* F4 challenge significantly reduced the expression of the three intestinal integrity markers involved in the organization of tight junctions. On the contrary, many of the treatments improved the expression of the three considered genes, even if at different extents. ZO-1 expression was ameliorated by GEO (+32%), while ZO-2 levels were enhanced by CapOR (+47%); ZnO increased mRNA levels of the two markers. Conversely, while ZnO did not improve CLD-1 expression, both CapOR and TTO significantly increased CLD-1 (+32%) compared to CTR+. ThyEO could numerically improve the levels of the three tight junctional markers, even if differences were not significant.

Despite not substantially ameliorating the expression of ZO-1, ZO-2, and CLD-1, GSE significantly decreased TNFα (−32%), IL-1β (−40%), and IL-8 (−50%), counteracting their dramatic activation when an *E. coli* F4 challenge was applied to Caco-2 cells. TNFα levels were also reduced by ThyEO (−37%) and CapOR (−40%), while ZnO tended to decrease its expression as well. On the other hand, TTO, ZnO and, to a lesser extent, ThyEO, increased the mRNA amount of IL-8 compared to CTR+, while ZnO also significantly augmented IL-1β levels.

Even though the bacterial challenge reduced the mRNA levels of BD1, ThyEO, CapOR, OE, ZnO, and GSE improved or restored its expression at the same value of the negative control.

### Adhesion assay

**Figure 5:**
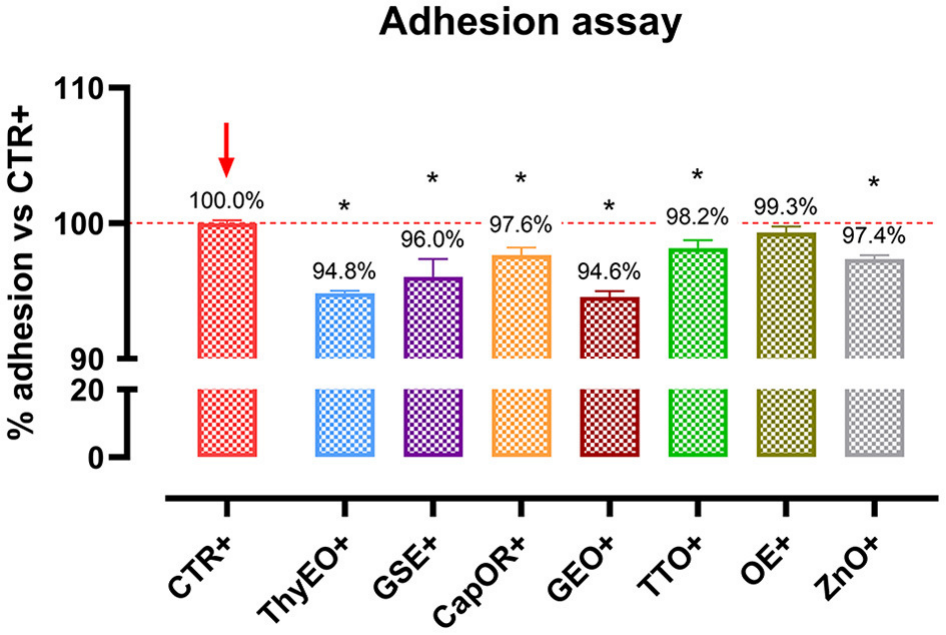
*Escherichia coli* F4 adhesion assay performed on Caco-2 cells treated with essential oils or powder extracts for 1 h. Groups with bacterial infection are represented with a “+” in the name. Data in the graphs are represented as means ± SEM. One-Way ANOVA analysis is performed against positive control (CTR+), identified with a red arrow; asterisks “*” denote significant differences with *p* < 0.05.

To investigate the ability of the employed essential oils and powder extracts to influence bacterial virulence by modulating their interaction with target cells, an adhesion assay was performed. Results are displayed by Figure 5. All the compounds significantly reduced the capacity of *E. coli* F4 to adhere to Caco-2 cells, except for OE. The most effective ones were ThyEO and GEO, followed by GSE. CapOR, ZnO, and TTO significantly reduced the adhesion of the pathogen as well, even if to a lesser extent.

### Immunofluorescence assay for ZO-1

**Figure 6:**
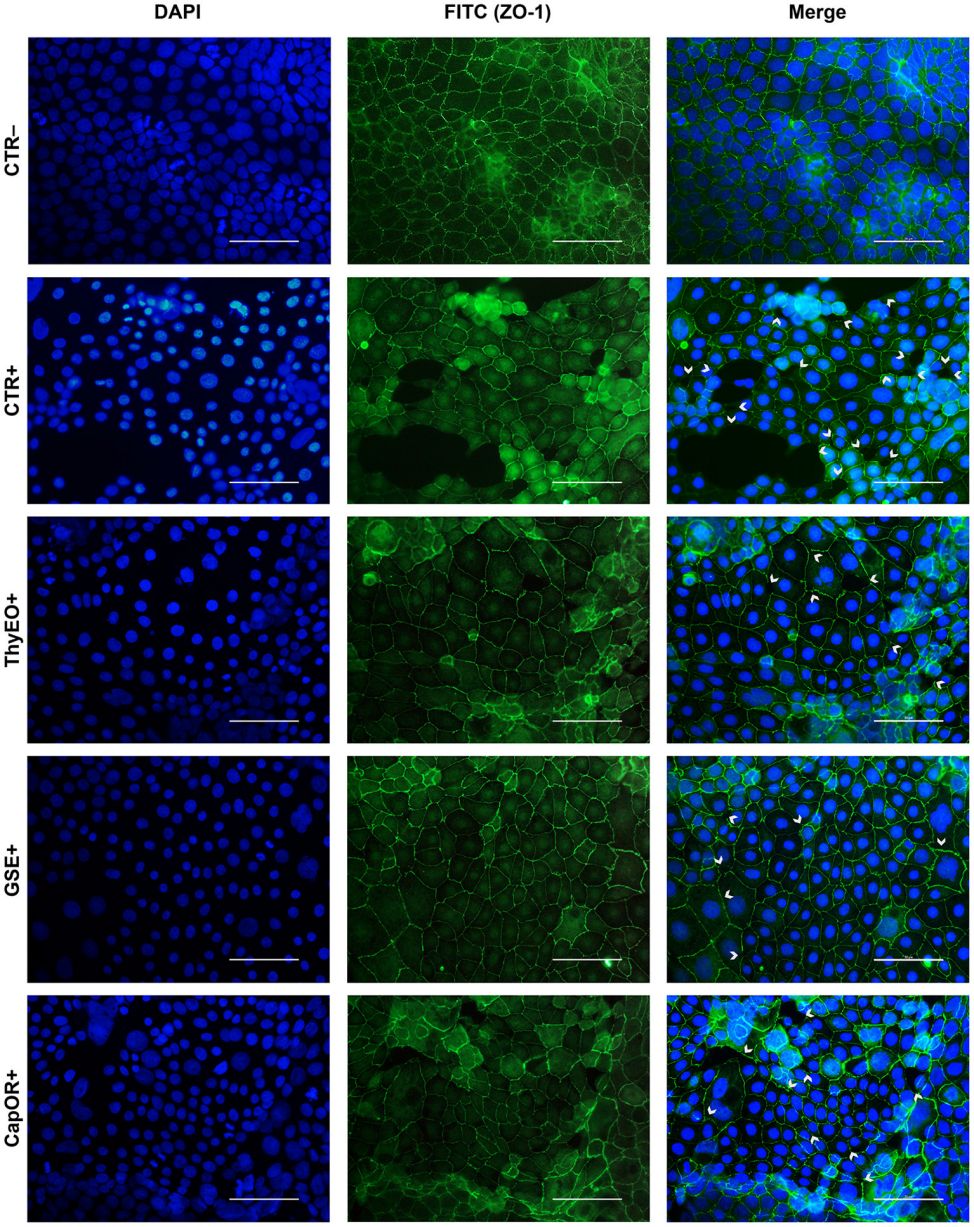
Immunofluorescence staining of Caco-2 cells treated with essential oils or powder extracts and simultaneously challenged with *E. coli* F4 for 2 h. Groups with bacterial infection are represented with a “+” in the name. In the image, the left column represents DAPI staining, the central column ZO-1 staining with FITC, while right column depicts the merge of the first two images, where white arrows identify areas of tight-junction detachment, loss of cells, holes in the monolayer or anomalies in ZO-1 disposition. Each row displays a different treatment.

**Figure 7:**
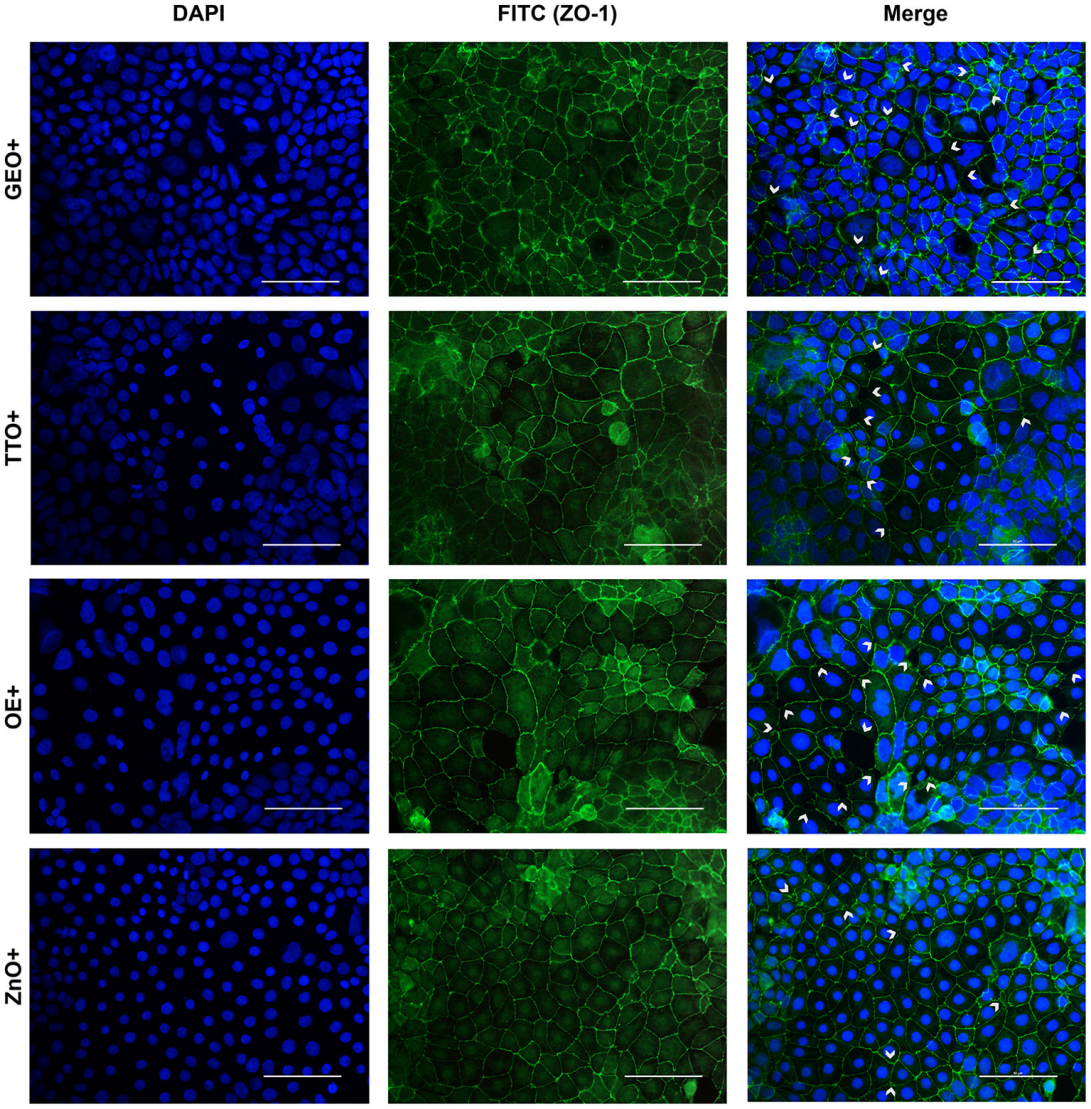
Immunofluorescence staining of Caco-2 cells treated with essential oils or powder extracts and simultaneously challenged with *E. coli* F4 for 2 h. Groups with bacterial infection are represented with a “+” in the name. In the image, the left column represents DAPI staining, the central column ZO-1 staining with FITC, while right column depicts the merge of the first two images, where white arrows identify areas of tight-junction detachment, loss of cells, holes in the monolayer or anomalies in ZO-1 disposition. Each row displays a different treatment.

The immunofluorescence staining for ZO-1 is shown in Figures 6, 7. The infection of Caco-2 with *E. coli* F4 produced significant damages on the cellular monolayer. In particular, intestinal epithelial cells suffered from ZO-1 loss of sealing capacity, producing open holes between adjacent cells. Moreover, bacterial challenge caused areas of significant mortality, with cells detaching from the surface of glass slides, generating acellular zones. Finally, in some areas, ZO-1 lost its correct localization, to form irregular agglomerates near cell borders, differing from the homogeneous and uniform pattern usually highlighted in unchallenged Caco-2 enterocytes.

Amongst treatments, ZnO significantly reduced the damages exerted by *E. coli* F4 infection: the integrity of Caco-2 monolayer was maintained, with only minor sites of ZO-1 misplacement or loss of tightness. Similar results were obtained with GSE, while ThyEO and CapOR, despite showing some areas of tight junction disruption, were generally effective in preventing profound damages or extensive losses of cell-to-cell contacts.

On the contrary, broader damages were observed for TTO and OE: a higher number of areas left opened between adjacent cells were found with TTO and OE treatments, together with zones of partial impairment in ZO-1 localization, even if not at the same extent of CTR+.

Despite showing a high degree of cellularity, without evident holes in the Caco-2 monolayer, treatment with GEO during the bacterial challenge did not protect cells from alterations to ZO-1 disposition along the cellular margins. In fact, sites of cell-to-cell contact loss were evident, together with frequent zones of ZO-1 abnormal localization, far from the common regular arrangement with sharp cellular borders.

## Discussions

Historically, pharmacological doses of ZnO were widely employed during the weaning phase of piglets to prevent the onset of PWD symptoms (34, 35). For a long time, it was believed that ZnO had a strong antimicrobial activity, but several studies demonstrated a remarkable action only at very high doses, principally against Gram-positive species (36, 37). Indeed, many *in vivo* trials reported how ZnO exerts mild effects on the fecal excretion of *E. coli* F4, the Gram-negative pathogen mainly responsible of PWD in piglets (38).

The ZnO mechanism of action seems rather related to a multifactorial effect on small intestinal enterocytes, the main target of ETEC infections (1). As confirmed by our results, ZnO protects cultured intestinal cells against *E. coli* F4 by maintaining a better epithelial integrity, thus reducing bacterial translocation across the cellular monolayer. The increased expression of selected tight-junctions, the modulation of inflammatory cytokines expression, and the enhanced levels of beta defensins, all contribute to explain the higher resilience that Caco-2 cells have when treated with ZnO during a bacterial challenge. Moreover, consistently with the outcomes reported by Roselli et al. (14), ZnO significantly reduced the count of viable *E. coli* F4 adhered to enterocytes, since it interferes with bacterial adhesins expression and function (39).

Despite their powerful activity, pharmacological doses of ZnO are no longer sustainable because of the risks related to their extensive use in animal husbandry (17). To overcome these issues, novel environmentally friendly alternatives to support piglets' weaning transition at the nutritional level are urgently needed. Natural extracts represent ideal candidates to substitute ZnO because the bioactive molecules they convey possess diverse mechanisms of action which modulate pathogens growth, while simultaneously supporting intestinal morphology and function (25, 32).

Maintaining intestinal integrity is a key factor to ensure piglets health and a smoother weaning transition: the loss of the gastrointestinal barrier elicits the establishment of a mild inflammatory status which results in the onset of diarrhea (40–43). Our infection model proved that, when *E. coli* F4 was incubated on Caco-2 cells with ThyEO, GSE, and CapOR, the damages of the challenge were significantly reduced, with TER levels in line with negative control. Thanks to the enhancement of the barrier integrity, a reduced bacterial translocation was also reported for the three extracts. Data are supported by the immunofluorescence staining of infected cells: a significant reduction of damages and an improved tight junction function was registered when ThyEO, GSE, and CapOR were added to the system, in agreement with ZnO. The positive effects of the three botanicals can be ascribed to their mechanisms of action.

Intestinal integrity is ensured by a complex system of sealing proteins that form tight junctions between adjacent cells and strictly regulate paracellular pathways (44). Two of the main actors are zonula occludens and claudins (45, 46). *Escherichia coli* F4 deeply affects the structure and functionality of these proteins: studies showed that ETEC toxins disrupt junctions by targeting ZO-1, CLD-1, and many cytoskeletal components (4, 7, 47). This is confirmed by our results: the pathogen impaired the expression of all the analyzed markers. However, when CapOR was added to the system, it increased the expression of CLD-1 and ZO-2, showing the ability to modulate two markers of intestinal integrity as occurs with ZnO.

Capsaicin, the main component of CapOR, transiently increases tight junction permeability without affecting their structure (48). This phenomenon is also reported by our data: at 2 h after the beginning of the infection, CapOR reported a lower TER, if compared to several other treatments. However, at 4 h, TER was maintained at a high level, demonstrating how the opening of the paracellular way is only temporary, and that it is reversed by an increase in tight junction expression (49, 50), as also displayed by the qPCR results. The CapOR transient loosening in epithelial integrity is not due to a disruption of cellular monolayer but, instead, it is tightly regulated (51–53): despite the slight drop in TER at 2 h, bacterial translocation still showed a reduction trend compared to CTR+.

The protective action of CapOR on tight junctions during *E. coli* F4 challenge is likely related to its main bioactive principle, capsaicin, the ligand of TRPV1 channels (54). Activation of TRPV1 by capsaicin reduced inflammation in LPS-challenged mice (55). Moreover, after binding TRPV1, capsaicin modulated the inflammatory activation in human endothelial cells treated with LPS, resulting in a decreased cytokine release (56). Consistently, our results show that CapOR controlled the upregulation of TNFα and IL-8 expression during an *E. coli* F4 challenge. This is in agreement with the findings of Alhamoruni et al., that confirmed how TRPV1 agonists protect Caco-2 intestinal cells from the increased permeability generated by high pro-inflammatory cytokines levels (57).

Slight numerical increases in tight junctions' expression and a modulation of TNFα was registered also for ThyEO during the *E. coli* F4 infection on Caco-2 cells. ThyEO is particularly rich in thymol, a bioactive compound recognized as a candidate agonist of TRPV1 (58) and a direct ligand of TRPV3 (59). Thymol's ability to interact with the endocannabinoid system and stimulate its anti-inflammatory potential could explain its ability to counteract ETEC negative effects. Thymol has also a direct action on the cellular inflammatory response, because its phenolic hydroxyl group interacts with NF-kB, a transcription factor involved in the response to stressful stimuli (60, 61) and associated with the MAPK pro-inflammatory pathway (62–64).

Other than modulating cellular inflammatory response, thymol deeply influences bacterial virulence genes expression (65, 66). Several studies demonstrated how thymol not only limits bacterial growth, but also controls *E. coli* F4 quorum sensing effectors, reducing ETEC ability to target intestinal cells (29, 67). This secondary mechanism of action against pathogens could explain why ThyEO did not exert extensive effects on Caco-2 cells, though keeping high TER values. Instead of exclusively acting on the intestinal mucosa side, thymol effect could be also oriented toward bacterial virulence, as previously demonstrated with *Salmonella typhimurium* (68). As a confirmation, thymol reduced the adhesion of the pathogen to Caco-2 cells at an extent higher than ZnO.

A similar effect is registered also for GSE, an extract rich in polyphenols. Traditionally, polyphenols were considered antinutritional factors because of their ability to bind proteins (69, 70). However, since many bacterial toxins are proteins, researchers have explored the impact of polyphenols against bacteria (71–73). Polyphenols are effective in inhibiting *Vibrio cholerae* CT toxin by preventing its cellular internalization (74–76). These results are of particular interest since CT toxin shares a high sequence homology to ETEC LT toxins (74, 77), and contribute to explain why GSE protected Caco-2 cells from the *E. coli* F4 infection at levels close to ZnO. Moreover, since polyphenol-rich extracts are able to control the expression of *E. coli* virulence as well (78), it is possible that the bacterial adhesion reduction in the GSE-treated Caco-2 cells is due to polyphenols' ability to interfere with ETEC quorum sensing systems.

Phenolic compounds from grape by-products embody a wide number of antioxidant molecules (79). Bacterial components such as LPS, after binding TLR4 receptors, induce an inflammatory response: the stimulation of transcription factors like NF-kB triggers oxidative enzymes and the uncontrolled accumulation of ROS (80, 81). In turn, ROS promote the inflammasome formation, creating a self-amplifying cycle that dramatically impairs cellular health (82). Grape by-products such as GSE directly detoxify ROS species and break this pro-inflammatory loop (83), improving cellular integrity, as highlighted by our results. The increased variability registered for this result might be due to the indirect effect that this extract exerts against the bacterial infection: the degree of activation of anti-virulence and anti-inflammatory responses relies on complex pathways that might produce a higher variability in the final response if compared to compounds that also possess more direct mechanisms of action such as the antimicrobial one, as happens for ThyEO.

Our study showed that GSE significantly downregulated TNFα, IL-1β, and IL-8 pro-inflammatory cytokines during *E. coli* F4 infection. This effect is due to the capacity of polyphenols to inhibit NF-kB by repressing the phosphorylation and elimination of IkB (84–86), the peptide that prevents NF-kB translocation into the nucleus. This is probably due to the scavenging ability of GSE polyphenols: by limiting ROS production, IkB phosphorylation is reduced, and NF-kB translocation is repressed (81). Furthermore, polyphenols activate Nrf2, a transcription factor that triggers several antioxidant enzymes (87). That is, polyphenols promote the degradation of the Nrf2 inhibitor Keap1 and increase Nrf2 nuclear translocation, revealing their dualistic effect in controlling both ROS production and the inflammatory cellular response (88, 89).

The supplementation of GEO, TTO, or OE did not significantly help cells against the *E. coli* F4 challenge. While some TER improvements were registered for TTO and OE at 2 h, the same were quickly lost at 4 h: the extracts were not able to maintain the positive effects during time. The results are confirmed by ETEC translocation, proving that structural damages to intestinal cells were not prevented. As a matter of fact, more irregular zones of ZO-1 distribution were observed in GEO, TTO, and OE groups, showing structural impairments to tight junctions. Their disposition, with ruffles and spikes, is in fact correlated with a higher epithelial permeability (90).

The reason why OE did not exert positive effects during the *E. coli* F4 infection is probably related to its main active principle, oleuropein. As an anticancer agent (91), oleuropein might dramatically exacerbate cellular apoptosis and autophagy, two natural tightly-regulated defense mechanisms of intestinal cells against ETEC (92), vanishing the mild antioxidant effect that the extract has (93). This hypothesis is corroborated by the high bacterial translocation and the loss of cells evidenced by the immunofluorescence staining of OE-infected enterocytes.

TTO and GEO are well known for their antioxidant and anti-inflammatory activity (94–97). However, our data demonstrate that, during an *E. coli* F4 infection, the two extracts could neither reduce cytokine expression, nor extensively restore tight-junction or defensin levels, except for TTO with CLD-1. It is possible that the experimental timeframe did not allow TTO and GEO to exert their beneficial effect on Caco-2 cells. Moreover, as previously demonstrated, their antioxidant potential is not as high as the one of ThyEO, GSE, or CapOR: during an oxidative challenge, TTO and GEO reduced ROS levels, but not at the same extent of the other extracts (28). This also depends on the exact composition of the essential oils employed, because different extracts harbor diverse bioactive principles at unique ratios.

Nevertheless, both GEO and TTO show interesting properties related to the control of *E. coli* F4 virulence (98–100). This is confirmed by our outcomes: even if they were not able to strongly help cells against the challenge, they still reduced bacterial adhesion to Caco-2 enterocytes, suggesting a potential modulation of ETEC adhesins. Further studies might be directed toward the confirmation of this mechanism of action and could also assess if GEO and TTO might be more effective as preventive treatments to prime intestinal cells before an infection.

## Conclusions

In conclusion, despite individual differences, ThyEO, GSE, and CapOR, were all effective in controlling *E. coli* F4 infection on intestinal enterocytes, showing peculiar mechanisms of action that mimic the ones of ZnO. While ThyEO had stronger effects on pathogen control itself and the production of defensins, GSE effectively mitigated the inflammatory response, and CapOR markedly re-established the expression of tight junctions, thus acting at many levels in the host-pathogen interaction. These outcomes support the utilization of botanicals to manage PWD in piglets, and future studies should now explore their effectiveness also *in vivo*. Furthermore, other researches could also evaluate the benefits of employing technologies like microencapsulation to ensure the delivery of unaltered botanicals' bioactive principles, preventing unwanted gastric modifications and enabling a more precise control of their inclusion in the feed. Other investigations might include the evaluation of their combination, to analyse if synergistic effects would enhance and complement the action of each single extract. Moreover, it would be interesting to explore how such botanicals could prepare cells to better react to *E. coli* F4, or to recover after a bacterial challenge.

## Data availability statement

The raw data supporting the conclusions of this article will be made available by the authors, without undue reservation.

## Author contributions

AB participated to the conceptualization of the research, performed the experiments, and wrote the original draft of the manuscript. EG conceptualized the research, participated during the investigation, reviewed the manuscript, and supervised the research. AP supervised the research. All authors approved the final version of the manuscript.
