# Supplementary material for: Botanicals as a zinc oxide alternative to protect intestinal cells from an Escherichia coli F4 infection in vitro by modulation of enterocyte inflammatory response and bacterial virulence
Source: Front Vet Sci. 2023 Mar 9;10:1141561. doi: 10.3389/fvets.2023.1141561 (PMC10033929; doi:10.3389/fvets.2023.1141561)
Supplement: Supplementary file 1 [file Data_Sheet_1.PDF]

Supplementary Material

1 Supplementary Figures

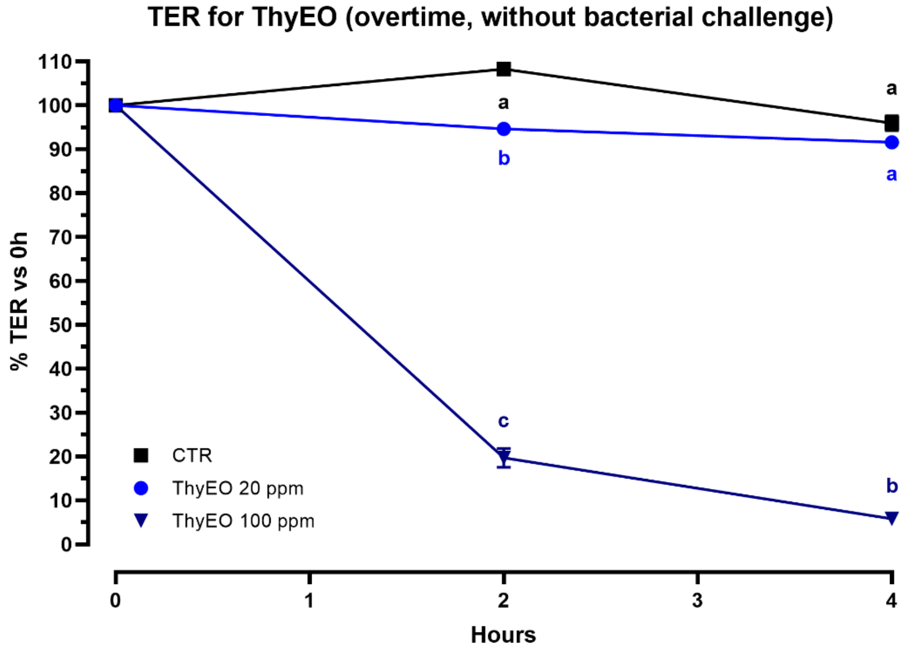

Supplementary Figure 1. TER of Caco-2 cells untreated (CTR) or treated with thymol essential oil (ThyEO groups) at 20 and 100 ppm for 4 hours. Data in the graphs are represented as means ± SEM; percentage values are referred to the initial TER value of each group before the beginning of the treatment, set at 100%. Two-Way ANOVA analysis is performed, with Tukey's multiple comparisons test between each group at any timepoint; different superscript letters denote significant differences with $p < 0.05$.

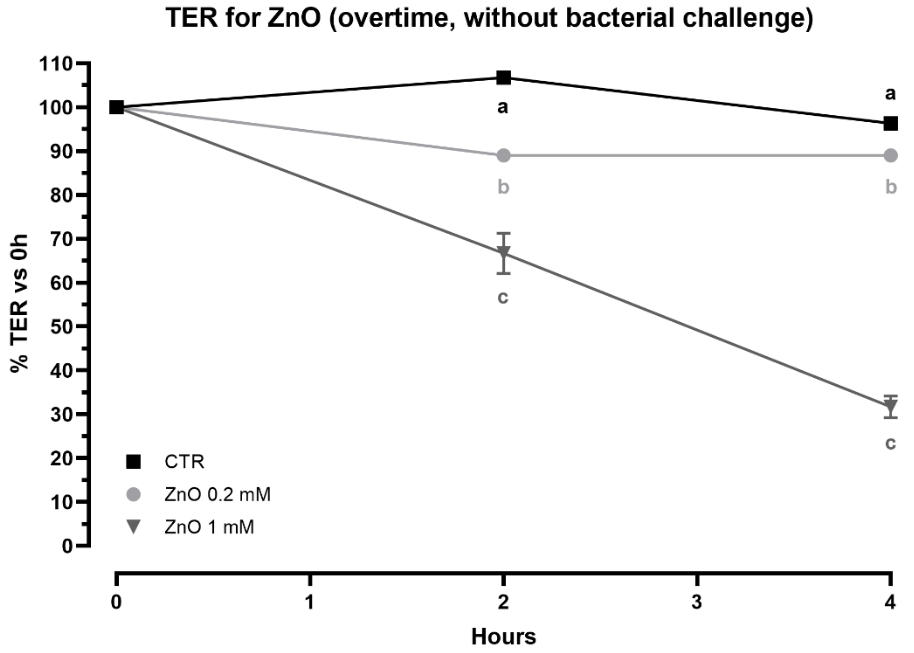

Supplementary Figure 2. TER of Caco-2 cells untreated (CTR) or treated with zinc oxide (ZnO groups) at 0.2 and 1 mM for 4 hours. Data in the graphs are represented as means ± SEM; percentage values are referred to the initial TER value of each group before the beginning of the treatment, set at 100%. Two-Way ANOVA analysis is performed, with Tukey's multiple comparisons test between each group at any timepoint; different superscript letters denote significant differences with $p < 0.05$.
